# Modulation of Rice Leaf Angle and Grain Size by Expressing *OsBCL1* and *OsBCL2* under the Control of *OsBUL1* Promoter

**DOI:** 10.3390/ijms22157792

**Published:** 2021-07-21

**Authors:** Seonghoe Jang, Jwa-Yeong Cho, Gyung-Ran Do, Yeeun Kang, Hsing-Yi Li, Jaeeun Song, Ho-Youn Kim, Beom-Gi Kim, Yue-Ie Hsing

**Affiliations:** 1 World Vegetable Center Korea Office (WKO), Wanju-gun, Jeollabuk-do 55365, Korea; yeeun.kang@worldveg.org; 2 Biotechnology Center in Southern Taiwan, Academia Sinica, Tainan 711, Taiwan; lihsingyi420@gmail.com; 3 Smart Farm Research Center, Korea Institute of Science and Technology (KIST), Gangneung, Gangwon 25451, Korea; chocho7023@naver.com (J.-Y.C.); hykim@kist.re.kr (H.-Y.K.); 4 Planning and Coordination Division, National Institute of Horticultural and Herbal Science, Rural Development Administration (RDA), Wanju-gun, Jeollabuk-do 55365, Korea; microdo@korea.kr; 5 Metabolic Engineering Division, National Institute of Agricultural Sciences, RDA, Jeonju 54874, Korea; icanje@korea.kr (J.S.); bgkimpeace@gmail.com (B.-G.K.); 6 Institute of Plant and Microbial Biology, Academia Sinica, Taipei 11529, Taiwan; bohsing@gate.sinica.edu.tw

**Keywords:** bHLH, cell elongation, leaf inclination, lamina joint, transcription factor, rice

## Abstract

Leaf angle and grain size are important agronomic traits affecting rice productivity directly and/or indirectly through modulating crop architecture. OsBC1, as a typical bHLH transcription factor, is one of the components comprising a complex formed with LO9-177 and OsBUL1 contributing to modulation of rice leaf inclination and grain size. In the current study, two homologues of *OsBC1*, *OsBCL1* and *OsBCL2* were functionally characterized by expressing them under the control of *OsBUL1* promoter, which is preferentially expressed in the lamina joint and the spikelet of rice. Increased leaf angle and grain length with elongated cells in the lamina joint and the grain hull were observed in transgenic rice containing much greater gibberellin A_3_ (GA_3_) levels than WT, demonstrating that both *OsBCL1* and *OsBCL2* are positive regulators of cell elongation at least partially through increased GA biosynthesis. Moreover, the cell elongation was likely due to cell expansion rather than cell division based on the related gene expression and, the cell elongation-promoting activities of *OsBCL1* and *OsBCL2* were functional in a dicot species, *Arabidopsis*.

## 1. Introduction

Rice leaf angle, the degree of bending between the leaf blade and culm, is a critical factor affecting plant architecture and grain yield [1,2]. In general, crops with erect leaves have increased photosynthetic efficiency and nitrogen storage for grain filling and are suitable for dense planting [3]. Many genes or *quantitative* trait loci (QTLs) such as *D61*/*OsBRI1*, *ILI1*, *LC2*, *ILA1*, *RAV6*, *OsARF19*, and *SLG* have been reported to control leaf angle [1,4,5,6,7,8,9]. Most rice mutants identified with altered leaf inclination have abnormal cell division and/or expansion and altered cell wall composition at the lamina joint [1,5,10,11]. In addition, phytohormones occupy an important place in the regulatory layers for rice leaf inclination [12]. In general, brassinosteroid (BR) affects grain size, leaf angle, and yield potential in rice. Moreover, leaf inclination is a distinctive BR-responsive architectural trait and BR-deficient or -insensitive mutants produce erect leaves in rice [4,13,14,15] while the exogenous application of BR or the genetic enhancement of BR signaling results in increased leaf inclination [16,17,18]. BR is known to affect cell elongation and/or cell division and both cell number and cell size are key factors mainly determining the size of each organ during plant development [19,20].

In rice, BR and gibberellin (GA) are two major phytohormones affecting plant height and leaf angle by regulating cell growth [21,22] and the crosstalk between the two phytohormones is mediated by complex networks; they interact at the signaling level as well as at the biosynthesis regulation level and the crosstalk can be distinct based on hormone concentrations, developmental stages, and different tissues even within a species [21,23]. For example, BR signaling mutants are impaired in GA biosynthesis [21] while GA regulates BR biosynthesis at the transcriptional level in rice. A negative regulator of the GA signaling pathway, *OsSPY* represses BR biosynthesis [13] and may also negatively regulate BR signaling by enhancing DELLA-BZR1 interaction [24]. Furthermore, a positive regulator of GA signaling, OsGSR1 activates BR synthesis through direct interaction with the BR biosynthesis enzyme, DWF1 [25]. *Oryza sativa Dicer-like 3a* (*OsDCL3a*) involved in the GA pathway has also been identified to regulate leaf inclination in rice [26] and impaired *OsDCL3a* expression by RNA interference caused increased leaf angle by modulating the expression of GA and BR associated genes, including *OsGSR1* and *BRD1*. Recent evidence emerging from a rice microRNA studies suggests that *OsmiR396d* also supports the notion that BR-GA co-regulation is implicated in leaf inclination [27].

Basic-helix-loop-helix (bHLH) proteins form the second largest family of transcription factors in plants, where they play key roles in critical metabolic, physiological and developmental processes [28]. In particular, 167 bHLH proteins have been identified in rice. These proteins can be divided into two groups, the atypical non-DNA-binding and the typical DNA-binding bHLH family based on to their DNA-binding activity [29], and they can be positive or negative growth regulators by interacting in an antagonistic and redundant manner to regulate various biological processes involved in growth including cell elongation, biosynthesis, stress resistance and signal transduction pathways [2,5,30,31,32,33,34].

It has also been reported that some bHLH proteins are involved in hormone biosynthesis and/or signaling in plants [2,34,35,36,37]. *Arabidopsis* PIF3, PIF4, PIF5, and PIF3-LIKE 5 (PIL5) are involved in the GA biosynthesis and signaling pathway [35,37] and the function of rice OsbHLH073 is associated with GA biosynthesis [34]. Other bHLH proteins play important roles in controlling BR signaling: *Arabidopsis* BEE1, BEE2, and BEE3, as products of early response genes are required for full BR response [35] and AIF2 interacts with BIN2 to participate in the BR signaling pathway [38]. In rice, INCREASED LAMINAR INCLINATION (ILI) and ILI1 BINDING bHLH1 (IBH1), regulate cell elongation in the lamina joint, affecting leaf bending in rice under BR induction [5]. Moreover, a trimeric complex formed by BRASSINOSTEROID UPREGULATED1-LIKE1 (OsBUL1), an atypical bHLH protein and OsBUL1 COMPLEX1 (OsBC1), a typical bHLH protein bridged by a small KxDL motif-containing protein, LO9-177 responds to BR signaling and regulates leaf inclination in rice [2]. Recently, *OsBLR1* (known as *OsbHLH079*) has been identified as a positive regulator of BR signaling for determining leaf angle and grain shape [11,39].

Here, we found that *OsBC-Like1* (*OsBCL1*, Os08g42470, *OsbHLH080*; [40]) and *OsBC-Like2* (*OsBCL2*, Os02g47660, OsBLR1, *OsbHLH079*; [11,39,40]), homologues of *OsBC1* (Os09g33580, *OsbHLH081*; [2,40]) function as positive regulators in cell elongation of laminar joints and grains in rice via, at least, increased GA biosynthesis. In spite of high sequence similarity between OsBCL1 and OsBCL2 at the protein level, distinct protein interaction patterns with putative partners are observed in the yeast two-hybrid system. Overexpression of *OsBCL1* and *OsBCL2* under the control of *OsBUL1* promoter driving lamina joint- and panicle-preferential gene expression, caused increased leaf angle and grain size. Furthermore, ectopic expression of the two genes in the dicot plant, *Arabidopsis*, resulted in narrow leaves with elongated epidermal cells confirming that *OsBCL1* and *OsBCL2* contribute to cell elongation in both monocot and dicot plants.

## 2. Results

### 2.1. Isolation of OsBCL1 and OsBCL2

**Figure 1:**
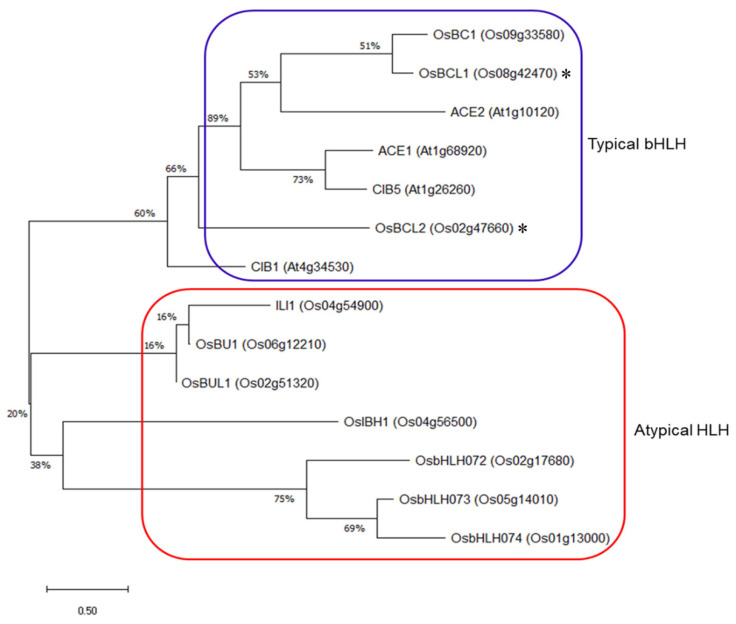
A phylogenetic tree showing the relationships among OsBC1, OsBCL1, and OsBCL2 proteins together with other typical bHLH (marked by blue line) and atypical HLH (marked by red line) proteins from *Arabidopsis* and rice. OsBCL1 and OsBCL2 are marked with an asterisk. The tree was constructed by the maximum likelihood method of MEGA X based on full-length amino acid residues. The percentage of replicate trees in which the associated taxa clustered together in the bootstrap test (1000 replicates) is shown next to the branches. Sequence information on each gene is available in the National Center for Biotechnology Information (NCBI) with an accession number.

OsBC1 is a transcription factor that plays a key role in determination of rice leaf angle by promoting cell elongation in the lamina joint. Based on the high sequence similarity to the OsBC1 at the protein level by using BLAST online search tools (https://blast.ncbi.nlm.nih.gov (accessed on 5 April 2018); https://www.ddbj.nig.ac.jp/index-e.html (accessed on 5 April 2018)), two rice genes encoding bHLH proteins were selected: *OsBCL1* (*OsBC1-Like1*) and *OsBCL2* (*OsBC1-Like2*). OsBC1 (OsBUL1 COMPLEX1) shared 57.8% and 28.0% identity with OsBCL1 and OsBCL2, respectively, in deduced amino acid sequence (Figure 1, Supplementary Table S1, https://www.uniprot.org/align/ (accessed on 5 April 2018)) and OsBCL1 is the closest homologue of OsBC1 in rice. Using the mixture of cDNAs synthesized from RNAs of various rice organs, the *OsBCL1* and *OsBCL2* clones containing full-length ORFs have been obtained by RT-PCR with the aid of gateway cloning system. OsBCL1 and OsBCL2 proteins consist of 291 and 361 amino acids, respectively, and like OsBC1, both OsBCL1 and OsBCL2 are putative transcription factors containing a typical basic helix–loop–helix (*bHLH*) domain in the middle of the proteins that encompasses residues 129–179 of OsBCL1 and residues 170–220 of OsBCL2, respectively (https://prosite.expasy.org/ (accessed on 5 April 2018)).

### 2.2. Increased Inclination Angle of Leaves Was Caused by Higher Expression of OsBCL1 and OsBCL2 in the Lamina Joint

**Figure 2:**
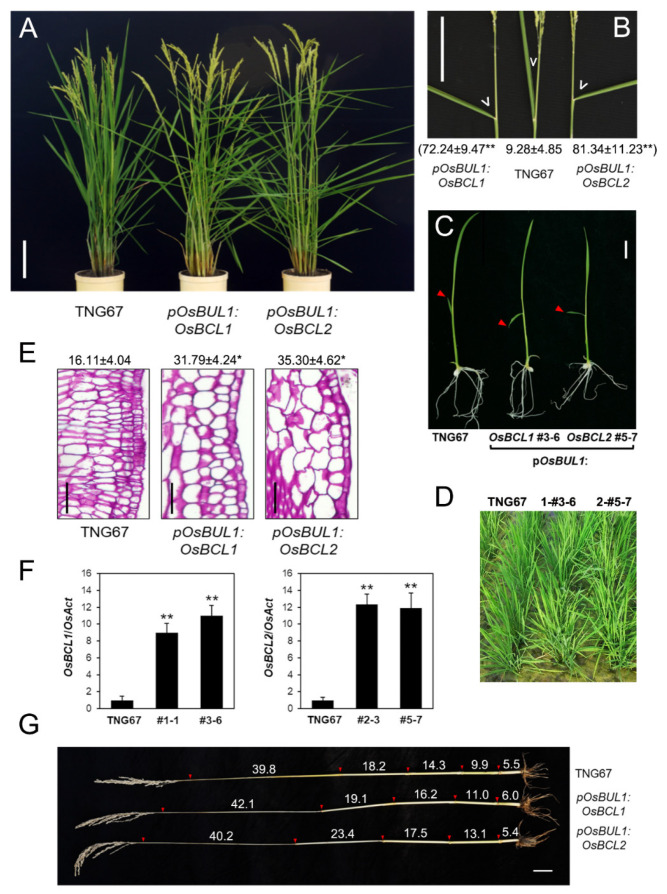
Phenotypic alterations of transgenic rice plants. (**A**) Transgenic rice harboring p*OsBUL1*:*OsBCL1* and p*OsBUL1*:*OsBUL2* constructs. Bar = 15 cm. (**B**) Leaf angles of the flag leaf (degree) after heading are marked by white arrow heads in transgenic rice plants, p*OsBUL1*:*OsBCL1* #1-1 and p*OsBUL1*:*OsBUL2* #5-7 together with wild type control, TNG67. Values are presented as means ± SD (degree; *n* > 10; **, *p* < 0.0001, Student’s *t* test). Bar = 10 cm. (**C**,**D**) Bending of the primary leaves (first seedling leaves—tips are marked by red triangles) and mature leaves of transgenic rice plants. Bar = 1 cm. (**E**) Histologic examination of the lamina joint of flag leaves. Length of cells in the lamina joint of p*OsBUL1*:*OsBCL1* and p*OsBUL1*:*OsBUL2* with TNG67 control plants is presented. Values are given as means ± SD (µm; length; *n* > 15; *, *p* < 0.01, Student’s *t* test). (**F**) Expression of *OsBCL1* and *OsBCL2* in 10-day-old transgenic seedlings containing p*OsBUL1*:*OsBCL1* and p*OsBUL1*:*OsBUL2*, respectively. Each bar represents mean ± SE of three independent experiments (**, *p* < 0.01, Student’s *t* test). (**G**) The length of each internode in transgenic plants (cm). Bar = 5 cm.

*OsBCL1* and *OsBCL2*, the two homologous genes of *OsBC1* were expressed under the control of *OsBUL1* promoter, which is preferentially active in the lamina joint and flower of rice. Compared to the WT control, transgenic rice plants containing p*OsBUL1*:*OsBCL1* and p*OsBUL1*:*OsBCL2* produced more inclined flag leaves, by 72.2° and 81.3°, respectively (Figure 2A,B,F). This phenotype was observed from the first leaf at the 2-week-old seedling stage (Figure 2C). The inclined leaves were continuously observed together with elongated lamina joints (Figure 2B–D; Supplementary Figure S1) through all the developmental stages, and may be due to elongated cells in the transgenic plants. Indeed, the cell length of the lamina joints in transgenic plants of p*OsBUL1*:*OsBCL1* and p*OsBUL1*:*OsBCL2* is greater than that of WT by 19.19 µm and 15.68 µm, respectively (Figure 2E). Furthermore, internode elongation was also found in transgenic plants (Figure 2G). Thus, it is likely that a higher level of *OsBCL1* and *OsBCL2* expression has a positive influence on the cell elongation at specific places where *OsBUL1* promoter is active.

### 2.3. GUS Expression Driven by OsBCL1 and OsBCL2 Promoters Exhibited Similar but Different Patterns in Rice

**Figure 3:**
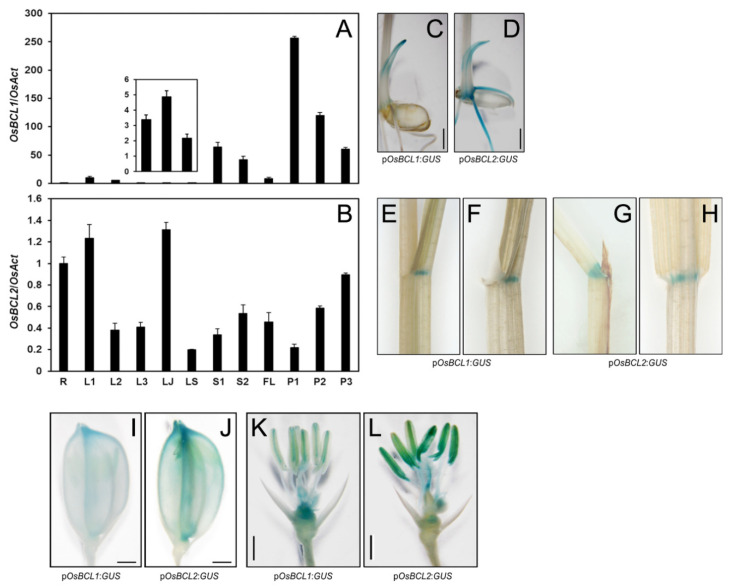
Spatiotemporal expression of *OsBCL1* and *OsBCL2*. (**A**,**B**) Relative expression level of *OsBCL1* and *OsBCL2* in various organs at different developmental stages. Magnified image of the *OsBCL1* expression level in mature leaves (L3), lamina joint (LS) and leaf sheath (LS) is shown in the box (**A**). Expression level of each gene was determined by qRT-PCR analysis and normalized to that of *OsAct*, showing mean ± SE (*n* = 3). R, 4-week-old roots; L1, 4-week-old leaves; L2, 50-day-old leaves; L3, 100-day-old leaves; LJ, lamina joint from 100-day-old leaves; LS, leaf sheath from 100-day-old plants; S1, stem from 50-day-old plants; S2, stem from 100-day-old plants; FL, flag leaves; P1, panicle length < 5 cm; P2, panicle length < 10 cm; P3, panicle length 10–15 cm. (**C**–**L**) GUS staining of various tissues from p*OsBCL1*:*GUS* and p*OsBCL2*:*GUS* transgenic rice plants, seedlings (**C**,**D**; Bar = 2 mm), lamina joint (**E**–**H**), spikelets (**I**,**J**; Bar = 1 mm) and spikelets after removing palea and lemma (**K**,**L**; Bar = 1 mm).

To investigate the spatiotemporal expression of *OsBCL1* and *OsBCL2*, quantitative RT-PCR (qRT-PCR) was utilized using cDNAs synthesized from RNAs of various organs. Transcripts of *OsBCL1* and *OsBCL2* were detected in various organs (Figure 3A,B). Of interest, the expression patterns of the two genes were opposite in the developing stems (S1 and S2) and panicles (P1, P2, and P3) as rice plants become mature. Moreover, their spatial expression was also visualized by promoter-GUS expression assays. The 2.45 kb-long nucleotide sequence of the *OsBCL1* promoter region led to GUS expression at the tip of coleoptile, lamina joint, palea, lemma, lodicules, anthers, carpel, receptacle and rudimentary glume (Figure 3C,E,F,I,K) while 2.4 kb-long nucleotide sequence of the *OsBCL2* promoter led to GUS expression at the coleoptile, roots, lamina joint, palea, lemma, lodicules, anthers and stigmas (Figure 3D,G,H,J,L). Collectively, these results indicate that OsBCL1 and OsBCL2 may act in a redundant manner but also play a spatiotemporally specific role in cell elongation.

### 2.4. Elongated Grains Were Produced by the Increased Expression of OsBCL1 and OsBCL2 in Rice Flowers

**Figure 4:**
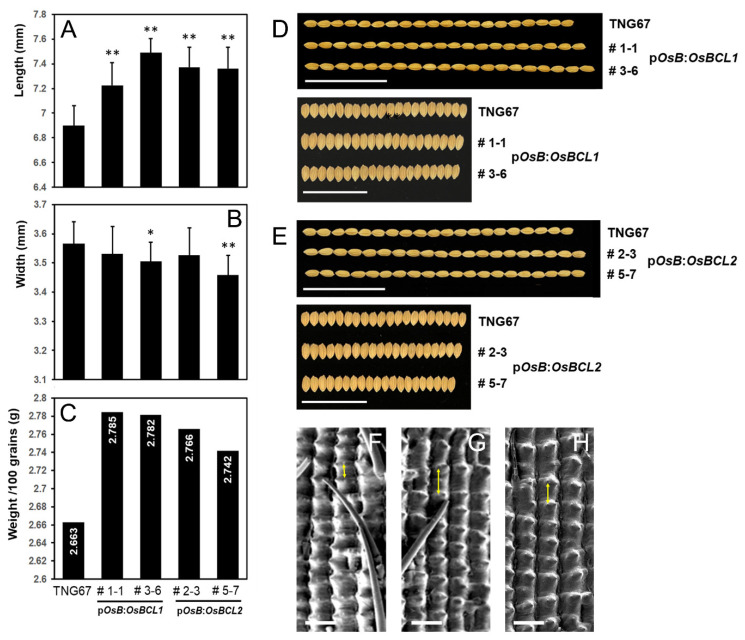
Grain size and weight. (**A**,**B**,**D**,**E**) Length and width of rice grains from transgenic rice plants with p*OsBUL1*:*OsBCL1* and p*OsBUL1*:*OsBUL2* constructs (*n* > 45; *, *p* < 0.05, **, *p* < 0.01, Student’s *t* test). (**C**) Weight of grains gained from transgenic and WT rice plants. (**F**–**H**) Epidermal cells of grains produced from p*OsBUL1*:*OsBCL1* (**G**), p*OsBUL1*:*OsBUL2* (**H**) and WT (**F**) rice plants. Distance between cells is marked by a two-way arrow. Bar = 100 µm.

Transgenic rice plants containing p*OsBUL1*:*OsBCL1* and p*OsBUL1*:*OsBCL2* produced grains with increased length and decreased width (Figure 4A,B,D,E), which is likely due to elongated epidermal cells in rice grain (Figure 4F–H). Moreover, increased weight of 100 grains gained from each transgenic plant was also recorded compared to the WT control indicating the larger grains are responsible for the heavier weight (Figure 4C). Thus, induction of expression of *OsBCL1* and *OsBCL2* in spikelets leads to the production of larger grains with elongated epidermal cells.

### 2.5. Both OsBCL1 and OsBCL2 Are Nuclear Proteins and Interact with OsIBH1, a Negative Regulator of Cell Elongation in Rice

**Figure 5:**
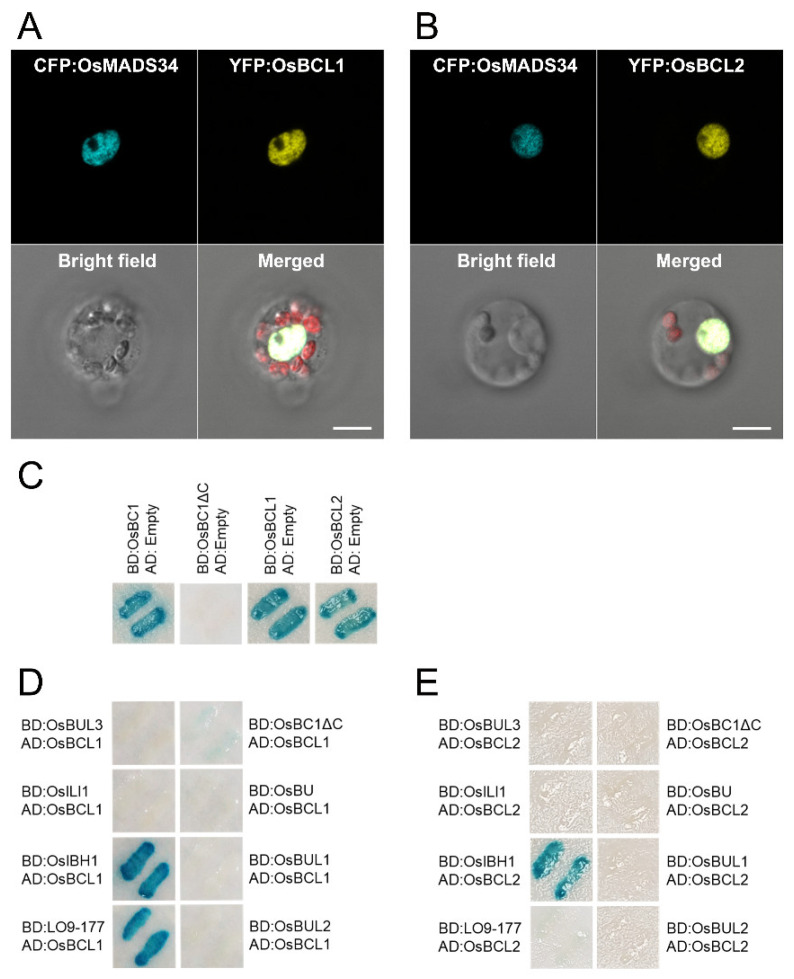
Subcellular localization and protein interaction partners of OsBCL1 and OsBCL2. (**A**,**B**) *YFP*:*OsBCL1* and *YFP*:*OsBCL2* were co-transformed into rice protoplasts respectively with *CFP*:*OsMADS34*, which is a nuclear marker. Bar = 10 µm. (**C**) OsBCL1 and OsBCL2 have auto-transcriptional activation activity. (**D**,**E**) Protein interaction partners of OsBCL1 and OsBCL2. BD and AD present GAL4 DNA binding domain and activation domain, respectively. Full-length form of each protein was fused to BD/AD domain of pBD/AD vectors and introduced into yeast cells and the interaction between two proteins was tested by x-gal filter assays.

The two chimeric proteins, YFP:OsBCL1 and YFP:OsBCL2, were localized in the nucleus, which was verified by the co-localization of a rice transcription factor, OsMADS34 in rice protoplasts (Figure 5A,B). As bHLH domain-containing nuclear proteins, both OsBCL1 and OsBCL2 exhibited transcriptional activation activity in the yeast system (Figure 5C). In addition, OsBCL1 strongly interacted with OsIBH1, a negative regulator of cell elongation and LO9-177, a component of the trimeric complex consisting of OsBUL1, LO9-177, and OsBC1, indicating the interaction pattern of OsBCL1 is similar to that of OsBC1 (Figure 5C). Furthermore, the formation of OsBC1-OsBCL1 heterodimers was also detected through yeast two-hybrid approaches (Figure 5D). In the case of OsBCL2, however, OsBCL2 could interact only with OsIBH1 under the detection sensitivity level of the yeast two hybrid system (Figure 5E), demonstrating distinct characteristics available between OsBCL1 and OsBCL2 in specific interaction with partners.

### 2.6. More GA_3_ Was Detected in the Transgenic Rice with Higher Expression of OsBCL1 and OsBCL2

**Figure 6:**
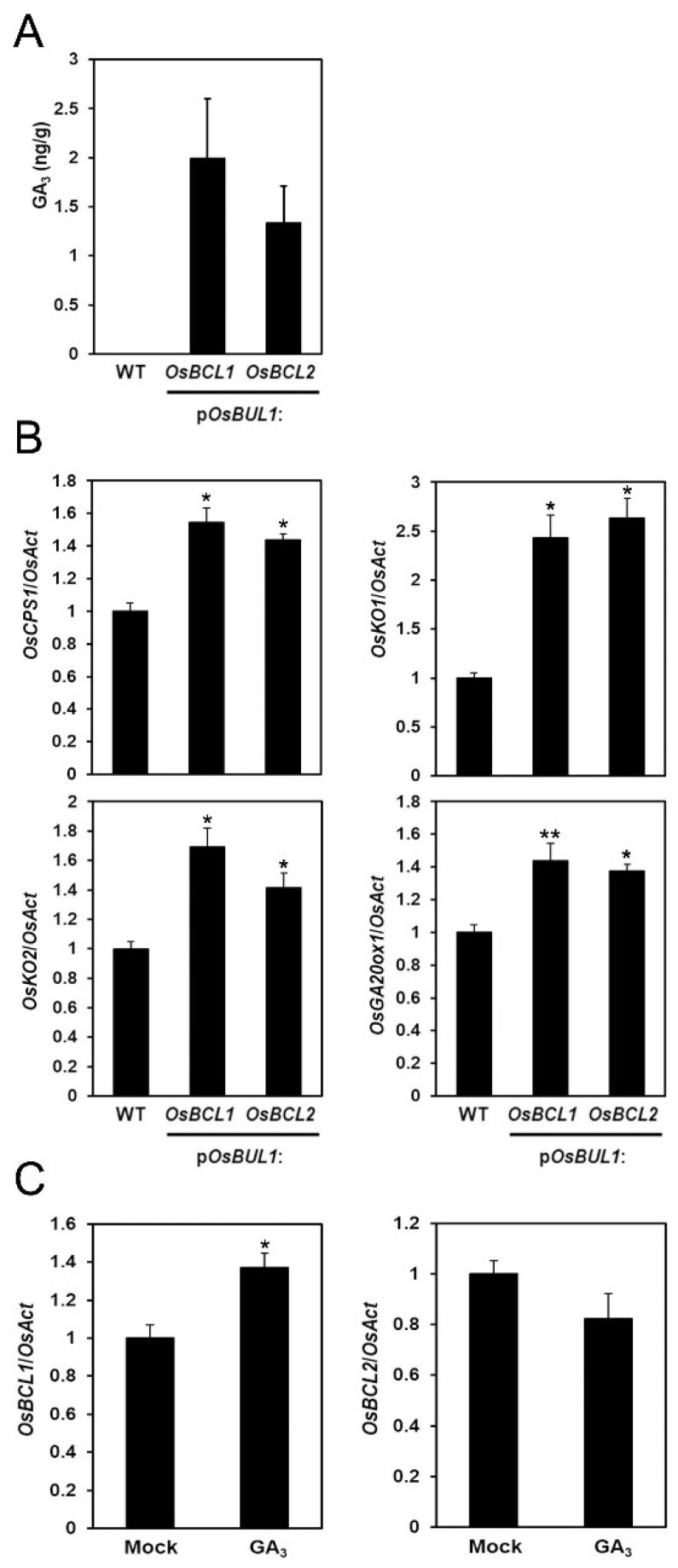
The content of GA_3_ increased in the transgenic rice plants. (**A**) The content of GA_3_ in p*OsBUL1*:*OsBUL1* (#3-6) and p*OsBUL1*:*OsBUL2* (#5-7) plants. No GA_3_ was detected in WT. (**B**,**C**) Expression of GA synthesis genes in transgenic plants and altered expression of *OsBCL1* and *OsBCL2* by exogenous application of GA_3_. The transcript level of each gene was determined by qRT-PCR analysis and normalized to that of *OsAct*. Each bar represents mean ± SE of three independent experiments ((**B**,**C**); *, *p* < 0.05, **, *p* < 0.01, Student’s *t* test). Above ground parts of 16-day-old rice seedlings grown at 14 h L, 28 °C/10 h (**D**), 26 °C were used for analyses.

**Figure 7:**
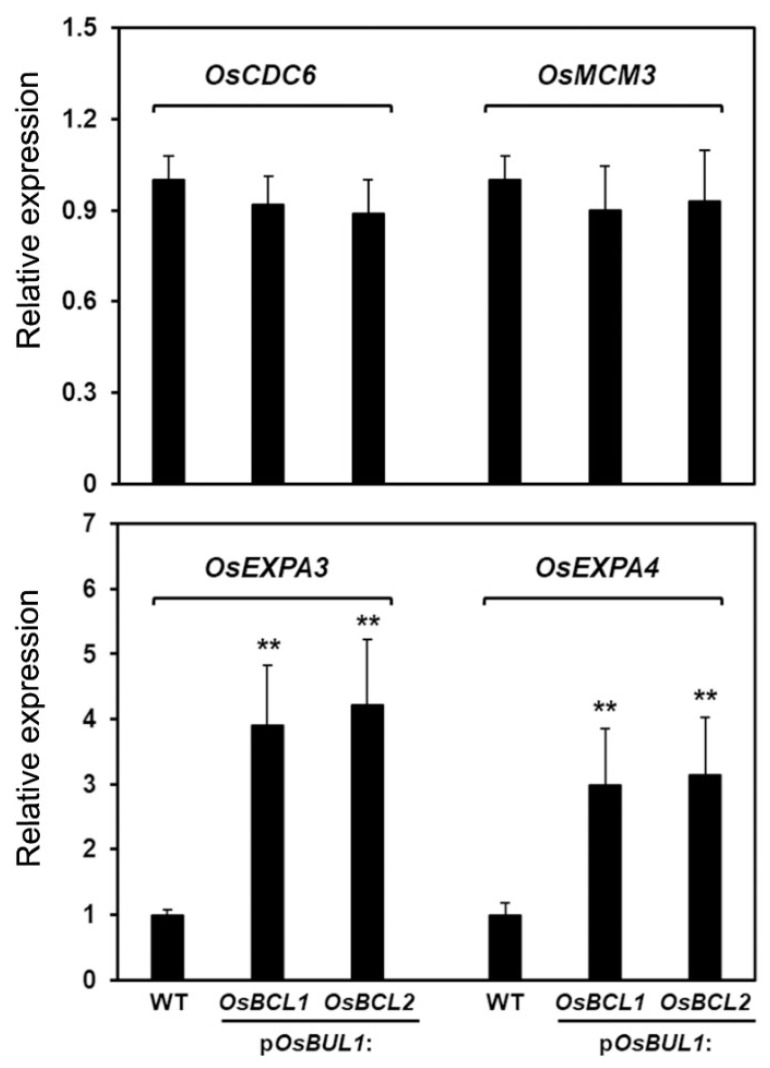
Expression of cell cycle- and cell elongation-related genes in transgenic rice plants. Expression levels of cell cycle-related genes, *OsCDC6* and *OsMCM3* (**upper**) and cell elongation-related genes, *OsEXPA3* and *OsEXPA4* (**lower**) in transgenic rice with p*OsBUL1*:*OsBCL1* and p*OsBUL1*:*OsBCL2* constructs compared to WT. Expression level of each gene was determined by qRT-PCR analysis and normalized to that of *OsAct*. Each bar represents mean ± SE of three independent experiments (**, *p* < 0.01, Student’s *t* test).

Because of the increased leaf inclination with elongated cells of lamina joint and grain epidermis in transgenic rice plants, we quantified the levels of phytohormones, such as gibberellin (GA_3_) and brassinosteroid (BL), to evaluate whether they are responsible for this phenotype in the transgenic plants, p*OsBUL1*:*OsBCL1* #3-6 and p*OsBUL1*:*OsBCL2* #5-7. As shown in Figure 6A, the amount of GA_3_ is dramatically increased in the transgenic plants while it is below detection level in the WT control indicating the increased leaf angle is linked to the higher amount of GA_3_ together with the higher transcript levels of transgenes and their endogenous expression in the lamina joint. However, BL was not detected in either the transgenic plants or the WT control, which is likely due to low level in the sample we used. Based on the increased amount of GA_3_ in the transgenic plants, we examined the expression of GA biosynthesis genes such as *OsCPS1*, *OsKO1*, *OsKO2*, and *OsGA20ox1*. The transcripts of these genes were found to be more abundant in the transgenic plants compared to WT (Figure 6B). In particular, *OsBCL1* expression was up-regulated by exogenous GA_3_ application although no significant difference in *OsBCL2* expression has been detected (Figure 6C). Furthermore, we speculated that the higher level of expression of *OsBCL1* and *OsBCL2* may affect cell division and/or cell expansion based on the erect leaf phenotype as several cases were observed especially in the cells of the lamina joint. As shown in Figure 7, expression of genes involved in cell expansion, such as *OsEXPA3* and *OsEXPA4*, is significantly higher in transgenic plants compared to WT control, whereas no alteration in the expression of genes for cell cycle regulation including *OsCDC6* and *OsMCM3* was observed, implying increased expression of the transgenes has an influence on cell proliferation (expansion) rather than cell cycle regulation.

### 2.7. Overexpression of OsBCL1 and OsBCL2 in Arabidopsis Causes Cell Elongation

**Figure 8:**
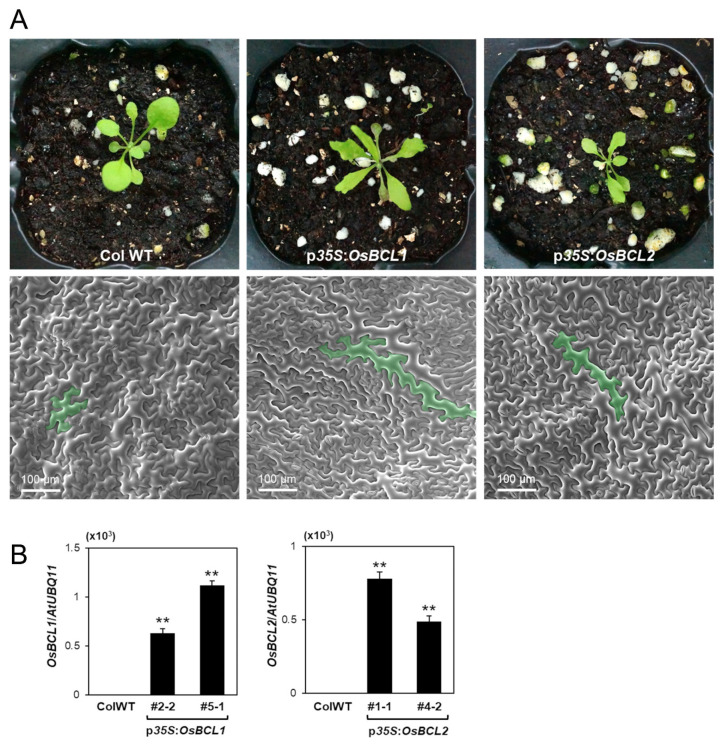
Overexpression of *OsBCL1* and *OsBCL2* in *Arabidopsis*. (**A**) Transgenic *Arabidopsis* plants with p*35S*:*OsBCL1* and p*35S*:*OsBCL2* exhibit narrow rosette leaves with elongated epidermal cells compared to WT. A representative epidermal cell from each genotype is marked in green. (**B**) Expression levels of transgenes, *OsBCL1* and *OsBCL2* in transgenic *Arabidopsis* were measured by qRT-PCR. Data represent mean ± SE of three independent experiments (**, *p* < 0.01, Student’s *t* test).

To examine the effect of *OsBCL1* and *OsBCL2* in dicot plants, constructs for overexpression of *OsBCL1* and *OsBCL2*, p*35S*:*OsBCL1* and p*35S*:*OsBCL2*, respectively, were generated and introduced into *Arabidopsis* via the *Agrobacterium*-mediated dipping method. Transgenic *Arabidopsis* plants overexpressing *OsBCL1* and *OsBCL2*, respectively produced elongated epidermal cells with narrow leaves implying that both *OsBCL1* and *OsBCL2* may have a positive effect on cell elongation in both monocot and dicot plants (Figure 8).

## 3. Discussion

In this study, we isolated two homologous genes of *OsBC1*, *OsBCL1*, and *OsBCL2*, in rice and characterized their expression and biological roles through functional studies. Ectopic expression of *OsBCL1* and *OsBCL2* under the *OsBUL1* promoter [2], which drives the gene expression to seedlings, lamina joints, nodes and panicles preferentially caused increased leaf angle in rice through the elongation of cells in lamina joints. In addition, increased length of internodes and grains has also been observed in transgenic rice plants. These phenotypic alterations are reminiscent of transgenic rice plants containing p*OsBUL1*:*OsBC1* [2]. Moreover, their spatiotemporal patterns of transcript abundance and GUS expression, particularly in the lamina joints and floral organs, are similar to the previous results gained from *OsBC1*. Thus, these results support the notion that *OsBC1*, *OsBCL1* and *OsBCL2* may play a critical and, at least, partially redundant role in cell elongation throughout rice development. In particular, the highest sequence similarity detected between OsBC1 and OsBCL1 at the protein level and their overlapping expression patterns strongly indicate that *OsBCL1* is the closest homologue of *OsBC1*. Based on the elongation of cells in the lamina joint and the lemma/palea in transgenic rice plants containing p*OsBUL1*:*OsBC1* and p*OsBUL1*:*OsBCL2*, endogenous levels of BR and GA_3_, two predominant phytohormones regulating plant cell elongation have been measured. A significant increase in GA_3_ was detected only in transgenic rice seedlings with p*OsBUL1*:*OsBCL1* and p*OsBUL1*:*OsBCL2*, while BR was not detectable in either WT or transgenic plants. It seems that the BR level in rice plants is below the limit of the detection system we used. Expression level of genes involved in GA biosynthesis was indeed higher in the transgenic seedlings compared to WT. Furthermore, expression level of genes involved in cell expansion including *OsEXPA3* and *OsEXPA4* was significantly increased in the transgenic plants compared to the WT whereas expression level of cell cycle genes such as *OsCDC6* and *OsMCM3* was indistinguishable from that of WT, supporting the notion that the larger leaf angles are mostly due to cell elongation/expansion rather than the increased number of cells in the lamina joint. Of note, however, only *OsBCL1* was upregulated by exogenous GA_3_ treatment for 24 h, indicating that distinct regulation of gene expression can be attained by exogenously applying GA_3_ among the three homologous genes, *OsBC1*, *OsBCL1* and *OsBCL2* [2]. Interestingly, a novel mechanism accounting for the negative regulation of rice leaf inclination was reported in a recent study showing that OsbHLH98, a typical bHLH transcription factor, counteracts the BR-induced cell elongation through transcriptional repression of *OsBUL1* encoding an atypical bHLH protein. Thus, based on the increased GA levels in the transgenic rice plants containing p*OsBUL1*:*OsBCL1* and p*OsBUL1*:*OsBCL2*, it will be worth investigating whether *OsBCL1*/*2* has a suppressive effect on the *OsbHLH073*, a negative regulator of GA biosynthesis containing an atypical bHLH domain, through GA-related pathways for cell elongation [34,41]. Protein-to-protein interaction patterns also support the distinct regulation of OsBCL1 and OsBCL2. OsBCL1 is able to interact with OsBC1 and LO9-177, which is a bridge molecule for the formation of the trimeric complex OsBUL1/LO9-177/OsBC1; however, OsBCL2 cannot interact with them although both OsBCL1 and OsBCL2 are nuclear proteins possessing transcriptional activation activity as OsBC1 homologues. Of note, OsBCL2 is known to interact with OsRACK1A (Receptor for Activated C-Kinase1), a member of RACK family containing the tryptophan-aspartate domain WD40. A recent report showed that the mutant rice impaired in the *OsRACK1* is shorter [42]. The function of *OsBCL2* has been intensively studied by two independent groups using activation tagging lines, overexpressing lines, dsRNAi lines and CRISPR/Cas9-mediated knockout lines [11,39]. *OsBCL2* is regarded as a putative upstream regulator of *REGULATOR OF LEAF INCLINATION1* (*RLI1*), encoding an HTH_MYB-like transcription factor, which activates leaf inclination by affecting the elongation of lamina joint cells through *OsBC1* [43]. Moreover, *OsBCL2* regulates leaf angle and grain shape by enhancing BR signaling pathway rather than controlling BR biosynthesis [11,39]. Gain- and loss-of-*OsBCL2* function caused increased and decreased leaf inclination and grain length, respectively, which supports our result gained from the ectopic expression of *OsBCL2* under the *OsBUL1* promoter. Further, elongated but reduced width of grains obtained from p*OsBUL1*:*OsBCL2* plants is coincident with the result from one of the groups using the *OsBCL2*-activation tagging line [11]. For practical application, suppressing the expression of *OsBC1*/*OsBCL1*/*OsBCL2* through dsRNAi approaches under the control of lamina joint-specific promoters may confer a reduced leaf angle phenotype on the rice plant without compromising any beneficial agronomic traits. Interestingly, OsBC1, OsBCL1 and OsBCL2 are able to interact with an atypical HLH protein, OsIBH1, a negative regulator of cell elongation in rice, implying there may be many layers of regulation in cell elongation through interacting networks of atypical HLH and typical bHLH proteins. Elongation of epidermal cells in transgenic *Arabidopsis* overexpressing *OsBCL1* or *OsBCL2* indicates that both *OsBCL1* and *OsBCL2* can play a positive role in cell elongation of dicot plants, showing functional validation of the two rice bHLH transcriptional factors in *Arabidopsis* and rice.

## 4. Materials and Methods

### 4.1. Plant Materials and Growth Conditions

Japonica rice (*Oryza sativa* L.) variety Tainung67 (TNG67) and *Arabidopsis* Columbia-0 (Col-0) were used as wild type. TNG67, a photoperiod insensitive flowering rice cultivar was used to produce transgenic rice plants and the transgenic plants were grown in a growth chamber (14 h L, 28 °C/10 h D, 26 °C) for 2 weeks after germination and moved to the outdoor GMO greenhouse of the Academia Sinica Biotechnology Center in Southern Taiwan and National Institute of Agricultural Science (NIAS), RDA-Korea. Transgenic plants used for analyses in this work are all T3 independent homozygous lines. For lamina angle measurement, ImageJ software (https://imagej.nih.gov/ij/docs/tools.html (accessed on 11 December 2019)) was used with photo files containing a rice leaf with its stem [44]. In general, *Arabidopsis* plants (Col-0) were grown in the growth chamber under LD conditions (16/8-h photoperiod at 100 μmol m^−2^ s^−1^) at 22 °C.

### 4.2. GA_3_ Treatment

Rice seedlings were germinated and incubated on MS media for 8 days and transferred to test tubes containing water (mock) or gibberellin (100 µM GA_3_ from Sigma Aldrich, St. Louis, MO, USA) solution, as described previously [2]. Whole parts above roots were harvested for RNA extraction at the 24 h time point after treatment.

### 4.3. Vector Construction and Plant Transformation

For p*OsBUL1*:*OsBCL1* and p*OsBUL1*:*OsBCL2* constructs, *OsBCL1* and *OsBCL2* entry clones were inserted into p*OsBUL1*:gateway (GW) vector [2]. Vector pGA3383 was used for analyzing promoter activities of *OsBCL1* and *OsBCL2* using the GUS reporter in rice [45]. Constructed plasmids were individually transformed into embryonic calli of TNG67 rice cultivars by *Agrobacterium tumefaciens*-LBA4404 mediation as described previously [46]. pGA643 vector was used for *Arabidopsis* transformation by floral dipping via *A. tumefaciens*-mediated DNA delivery [47]. More than 15 independent transgenic plants were initially generated and analyzed, and at least two independent homozygous lines were selected for phenotypic description.

### 4.4. RNA Extraction and Expression Analyses

Total RNAs from plant materials were extracted using an RNeasy Plant Mini Kit (Qiagen, Hilden, Germany) and treated with RNase-free DNase (Qiagen, Hilden, Germany) following the manufacturer’s protocol to remove any residual genomic DNA. DNase-treated RNA was subjected to reverse transcriptase reactions with oligo (dT) primer and Superscript III reverse transcriptase (Invitrogen, Carlsbad, CA, USA) based on the manufacturer’s protocol. Subsequent PCR was performed with the first-strand cDNA mixture and EX-Taq polymerase (Takara, Dalian, China). qPCR was conducted on a CFX96TM real-time system (Bio-Rad, Irvine, CA, USA) using Maxima SYBR Green qPCR Master Mix (Thermo, Waltham, MA, USA). The primers used for quantification are listed in Supplementary Table S2. For PCR, each sample was analyzed in triplicate. The run protocol was: denaturation at 95 °C for 10 min and annealing/extension repeated 45 times (95 °C for 15 s and 60 °C for 30 s, data acquisition was performed). Housekeeping genes such as *OsAct* [48] and *AtUBQ11* [49] was included in the reactions as internal controls for normalizing the variations in the amount of cDNA used. The threshold cycle (C_T_) was automatically determined for each reaction by the system set with default parameters.

### 4.5. GUS Staining

For promoter analyses, about 2.45 kb of *OsBCL1* and about 2.4 kb of *OsBCL2* 5′-regions were amplified using primers 5′ GCCGGATCCAATGCAAAGGTTGATGCTTGACTAG 3′ (p*OsBCL1*-BamHI-F) and 5′ GCGTCTAGATGCTTTGCTGCTGCTGAACTCTCTG 3′ (p*OsBCL1*-XbaI-R), and 5′ GCCGGATCCGCTACTTGCATGGACGTTGCGAC 3′ (p*OsBCL2*-BamHI-F) and 5′ GCCGGTACCATTACTAACCACTTAGCAAGAAGAG 3′ (p*OsBCL2*-KpnI-R), respectively, and cloned into pGA3383 vector for GUS fusion [45]. The resulting plasmids were transformed into rice, and GUS staining was performed according to the method described previously [50].

### 4.6. Phytohormone Sample Preparation

The extraction protocol was applied to the rice samples as described by Pan et al. (2010) with mild modification [51]. Samples were frozen and ground in liquid N_2_ right after harvesting, and 50 mg of the fresh plant tissues were extracted with solvent mixture (isopropanol:water:HCl = 2:1:0.002, *v*/*v*/*v*) by shake incubator at a speed of 100 rpm for 30 min in ice. Extracts were fractionated with dichloromethane 1 mL via shaking for 30 min and centrifuged at 13,000 g for 5 min at 4 °C. Supernatants were filtered through Chromafix C18 cartridge and concentrated by nitrogen evaporator (Allsheng MD 200, Hangzhou Allsheng Instrument Co. LTD, Hangzhou, China). Concentrates dissolved in methanol were filtrated by 0.22 µm membrane filter (PVDF syringe filter, hydrophobic, 13 mm diameter, 0.22 μm pore size, Whatman International, Maidstone, UK) before analysis. Phytohormones, standards gibberellin A3 (GA_3_; Sigma G7645) and brassinolide (BL; CAS No. 72962-43-7) were purchased from Sigma Aldrich (St. Louis, MO, USA) and Cayman Chemical (Ann Arbor, MI, USA), respectively.

### 4.7. UPLC-MS Analysis

The phytohormones were analyzed using UPLC-MS (Shimadzu LCMS-2000, Kyoto, Japan). Extracted samples (10 μL) were loaded to YMC-Triart C18 ExRS (100 × 2.0 mml.D. S-1.9 μm, 8 nm) maintained at 45 °C at 0.25 mL/min flow rate with solvents: (A) water containing 0.2% formic acid and (B) acetonitrile containing 0.2% formic acid. Gradient system was as follows: 0 min 0% B; 1 min 0% B; 3 min 30% B; 17 min 60% B; 19 min 0% B; 20 min 0% B. Quantitative analysis was carried out under the selective ion monitoring (SIM) mode based on calibration curve of standards. MS scan range was 120–550 *m*/*z* and, scan speed was maintained at 883 μ/s. Nebulizing gas and drying gas flows were set at 1.5 L/min and 15 L/min, respectively. 

### 4.8. Histological Analyses and Microscopy

The procedures were modified from a previously reported method [52]. Tissues were fixed in 2.5% glutaraldehyde (*v*/*v* in a 0.1 M phosphate buffer) at pH 7.2 in the presence of 4% sucrose (*w*/*v*) for 24 h. After three rinses (30 min, each) with the above buffer, the specimens were post-fixed with 1% OsO4 *w*/*v* in the same buffer with 4% sucrose (*w*/*v*) for 4 h. They were then rinsed three times (30 min, each) with the buffer, dehydrated in the alcohol series, transferred to propylene oxide and embedded in Epon epoxy resin. Semi-thin sections (2.5 µm) prepared by an ultra-microtome were collected on glass slides and the Periodic Acid–Schiff (PAS) polysaccharide specific reaction was carried out. PAS-positive reaction shows red color. Sections for staining were first plunged in 1% periodic acid (*w*/*v*) for 30 min, then in Schiff’s reagent for 40 min and finally in 5% sodium bisulfite (*w*/*v*) for 35 min. Sections were then rinsed in distilled water, dried on a warm plate, and mounted in Histomount for observation with a light microscope (Axioscop 2, Carl Zeiss, Oberkochen, Germany). In order to examine the morphological characters of epidermal cells, live tissues were examined on a SEM (SU-3500, Hitachi, Tokyo, Japan) operating at low vacuum mode.

### 4.9. Subcellular Localization of Proteins

For cellular localization of OsBCL1 and OsBCL2 in rice, yellow florescence protein (YFP):GW vector was used for the florescence fusion as described previously [53]. Subcellular localization of YFP:OsBCL1 and YFP:OsBCL2 fusion proteins was observed in rice protoplasts together with a nuclear marker, CFP:OsMADS34 [54]. Isolation and transfection of rice protoplasts were followed as described by Zhang et al. (2011) and images of cells with fluorescence were taken by confocal microscopy (LSM 510 META NLO DuoScan, Carl Zeiss) [55].

### 4.10. Yeast Two-Hybrid Assays

*OsBCL1* and *OsBCL2* full-length ORFs were cloned in-frame into pBD- and pAD-GAL4 vectors (Stratagene; [2]) to generate pBD:OsBCL1, pBD:OsBCL2, pAD:OsBCL1, and pAD:OsBCL2 constructs. Interacting partners were prepared for yeast two-hybrid interaction assays and X-gal filter assays were performed as described previously [2].

## Data Availability

Data is contained within the article or supplementary material.

## Supplementary Materials

The following are available online at https://www.mdpi.com/article/10.3390/ijms22157792/s1.
