# Supplementary figures and images for: Modulation of Rice Leaf Angle and Grain Size by Expressing OsBCL1 and OsBCL2 under the Control of OsBUL1 Promoter
Source: Int J Mol Sci. 2021 Jul 21;22(15):7792. doi: 10.3390/ijms22157792 (PMC8346013; doi:10.3390/ijms22157792)

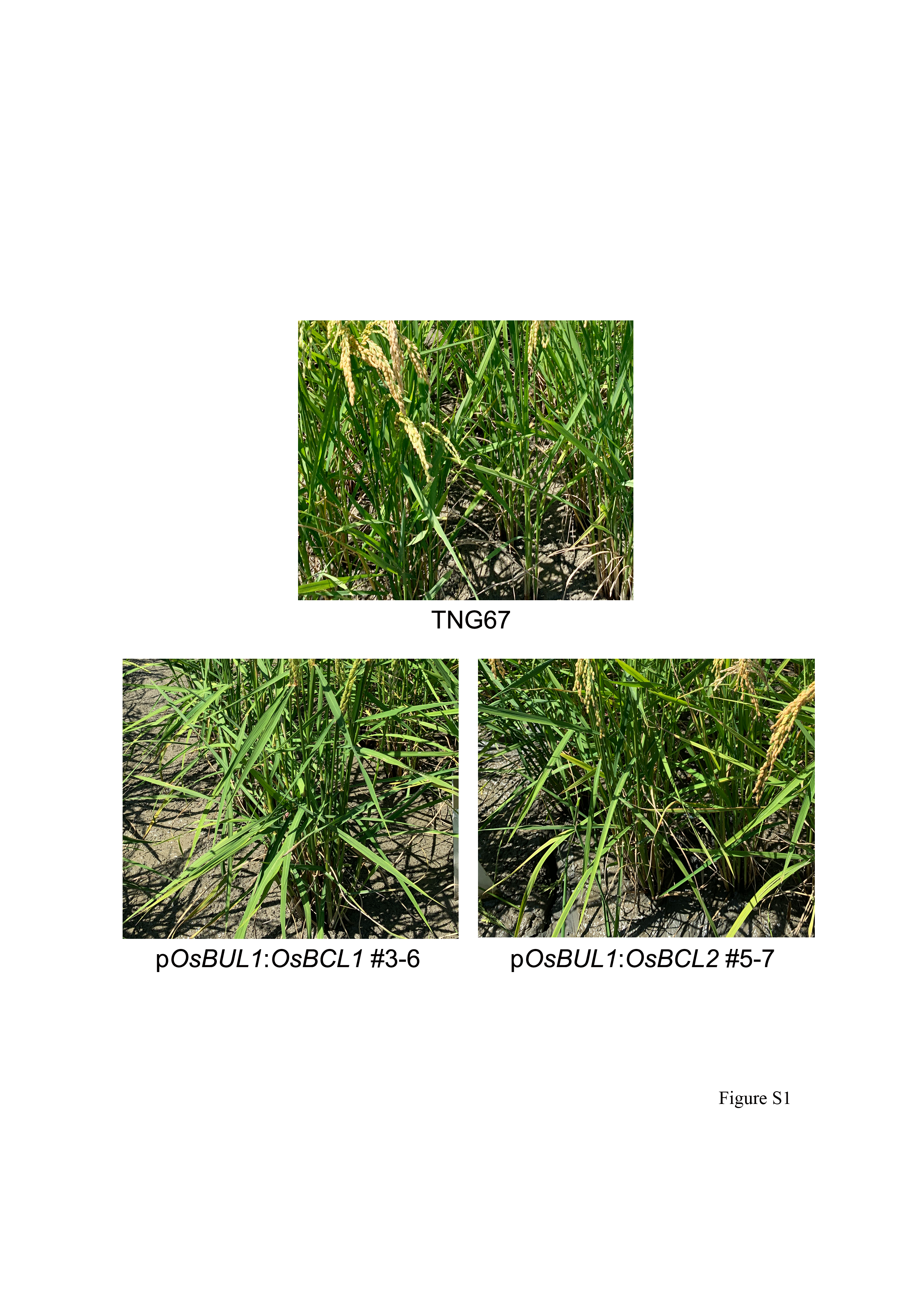

Supplement: Supplementary file 1 [file ijms-22-07792-s001.zip › Supp Figure S1 (July 16 2021).tif]
